# Integrating Metabolic Modeling and Targeted Supplementation for the Rapid Detection of *Clostridium tyrobutyricum* in Dairy Products

**DOI:** 10.3390/microorganisms14091999

**Published:** 2026-09-09

**Authors:** Idris Arslan

**Affiliations:** Faculty of Science Molecular Biology and Genetics, Zonguldak Bülent Ecevit University, Zonguldak 67100, Türkiye; idris.arslan@yahoo.com; Tel.: +90-3722911100

**Keywords:** *Clostridium tyrobutyricum*, late blowing defect, metabolic modeling, FBA

## Abstract

*Clostridium tyrobutyricum* is a major cause of late blowing defects (LBDs) in cheese, resulting in substantial economic losses. Early detection is critical for maintaining product quality. In this study, we developed a rapid detection approach integrating genome-scale metabolic modeling (GEM) with systematic culture optimization. Three media were evaluated, identifying RCM at 38.5 °C as the optimal condition for reducing the lag phase. Flux Balance Analysis (FBA) revealed that targeted supplementation with magnesium, zinc, Vitamin B6, and L-tryptophan significantly enhanced metabolic flux through nucleotide biosynthesis and energy transfer pathways, particularly reaction rxn01219_c0. Validation using artificially contaminated milk confirmed that the optimized 0.5× supplementation mixture synergistically reduced detection time by approximately 35 h compared to conventional MPN methods. This study demonstrates that bridging systems biology with traditional microbiology provides a cost-effective and mechanistic framework for rapid pathogen detection in the dairy industry.

## 1. Introduction

Foodborne diseases represent a significant and growing global public health concern, affecting more than 600 million people annually [1]. Foodborne pathogens include bacteria, viruses, fungi and parasites, which are responsible for human infections and pose a risk to both public health and the economy due to their adverse effects. Foodborne diseases are often caused by the consumption of food or water contaminated with pathogens and/or their toxins [2,3].

*Clostridium* is a large genus of obligate anaerobes belonging to the *Firmicutes* phylum of bacteria, the majority of which possess a Gram-positive cell wall structure. The genus contains significant human and animal pathogens, causative of potentially deadly diseases such as tetanus and botulism. The species of the genus *Clostridium* most widely involved in foodborne illness are *Clostridium perfringens* and *C. botulinum* [4].

*Clostridium tyrobutyricum* is a Gram-positive, strictly anaerobic bacterium, occurring alone or in pairs, which usually includes peritrichously flagellated and motile bacteria. It is rod-shaped and in the size range 1.9–13.3 × 1.1–1.6 μm. Spore structures are oval, subterminal, and swell the cell. The optimum growth temperature is in the range 30–37 °C, with moderate growth at 25 °C and poor or no growth at 45 °C. Surface colonies on anaerobically incubated blood agar plates are frequently β-hemolytic, circular, glossy, and gray, with a diameter of 0.5 mm. This microorganism has been isolated from raw milk and dairy products, chicken, chicken salad, fruit juices, silage, gley soil, and the fecal material of cattle, beagle dogs, and human adults and infants. *C. tyrobutyricum* is nonpathogenic to humans and animals. *C. tyrobutyricum* is characterized by intensive fermentation of carbohydrates to butyric acid and acetic acid as the main products, as well as CO_2_ and hydrogen (H_2_) as side products. Simple medium requirements for cell growth and its relatively high product purity and yield have made *C. tyrobutyricum* a microorganism of significant interest to different industries, including the chemical and pharmaceutical industries [5,6]. *C. tyrobutyricum* is a saccharolytic *Clostridium* spp that ferments lactate if acetate is also present in the growth medium, and hence, this carbon source is widely added to media to enhance detection of this organism. It produces only traces of alcohol in comparison to *C. tyrobutyricum*, which can produce significant amounts of butanol in the late stages of fermentative growth [6].

*C. tyrobutyricum* represents the major spoiling agent responsible for late blowing defects (LBDs) in hard and semi-hard cheeses. Its spores are also resistant to manufacturing procedures and can germinate during the long ripening process, causing the cheese paste to burst with a consequent undesirable taste [7]. This condition makes cheese unacceptable to the consumer.

Quantitative data on the exact percentage of dairy products rejected specifically due to *C. tyrobutyricum* are limited in the literature, as most studies report late blowing defect (LBD)-associated losses in qualitative terms (e.g., considerable or substantial economic losses) rather than as standardized rejection rates [8]. One of the few quantitative estimates comes from a predictive risk-assessment model developed for Gouda cheese, which simulated *C. tyrobutyricum* outgrowth using spore concentration data collected from eight dairy farms over a 12-month period. This model estimated that 9.2% (±1.7%) of cheeses exhibited LBD by day 60 of ripening, rising to 36.1% (±3.4%) by day 90 [9]. While these figures represent modeled predictions rather than direct industry rejection statistics, they nonetheless illustrate the scale of product loss attributable to this organism and underscore the practical importance of early, rapid detection methods such as the one proposed in this study.

The bacterial samples associated with the spoiled foods have been intensively studied by routine diagnostic methods, involving microbiological methods (most probable number (MPN) technique), molecular approaches (PCR-based methods and next-generation sequencing (NGS) method), and immunological techniques (immune assays and flow cytometry) [10,11,12]. In this framework, the development of a rapid, low-cost method that is effective in a short time is of importance.

Despite the availability of advanced detection methods such as PCR-based techniques and next-generation sequencing, these approaches often require specialized equipment, trained personnel, and relatively high operational costs. As a result, culture-based methods, particularly the most probable number (MPN) technique, remain widely used in routine dairy quality control due to their simplicity and reliability. However, a major limitation of these methods is the long incubation time required for detection, which delays decision-making in industrial processes. Therefore, there is a clear need for improved culture-based strategies that can significantly reduce detection time while maintaining practicality and cost-effectiveness.

To address this limitation, the objective of this study was not to replace rapid molecular diagnostics, but to develop an accelerated, cost-effective culture-based detection strategy. By coupling Flux Balance Analysis (FBA) predictions with systematic nutrient supplementation, we aimed to shorten the lag phase of *C. tyrobutyricum*, providing a practical tool for resource-limited dairy quality control laboratories.

## 2. Materials and Methods

### 2.1. Chemicals and Reagents

Reinforced Clostridial Medium (RCM), Brain Heart Infusion (BHI), and Fluid Thioglycollate Medium (FTM) were purchased from Merck KGaA (Darmstadt, Germany). All supplement compounds, including magnesium sulfate, zinc sulfate, pyridoxine hydrochloride (Vitamin B6), L-tryptophan, L-theanine, and piperine, were obtained from Sigma-Aldrich (St. Louis, MO, USA).

### 2.2. Bacterial Strain

*C. tyrobutyricum* ATCC 25755 was obtained from the Leibniz Institute DSMZ–German Collection of Microorganisms and Cell Cultures (Braunschweig, Germany). Until testing, the freeze-dried strain was reconstituted and stored as glycerol stocks (20% *v*/*v*) at −80 °C. Prior to experimental trials, working cultures were reactivated anaerobically at 37 °C in RCM. The strain was used for artificial contamination experiments to evaluate the performance of the proposed rapid detection method.

### 2.3. Culture Media and Growth Conditions

RCM, BHI, and FTM were used for cultivation and growth analysis of *C. tyrobutyricum*. All media were prepared according to the manufacturer’s instructions.

To determine the optimal growth conditions, bacterial cultures were incubated under anaerobic conditions at three different temperatures (37 °C, 38.5 °C, and 40.1 °C) across the three media. Growth performance was evaluated based on lag phase duration and overall growth dynamics.

### 2.4. Determination of Optimal Supplementation Conditions

To enhance bacterial growth and reduce detection time, selected organic and inorganic supplements were tested. Magnesium, zinc, Vitamin B6, L-tryptophan, L-theanine, and piperine were initially evaluated individually. Subsequently, a combined supplementation mixture (Table 1) was prepared and tested to assess potential synergistic effects on growth enhancement and detection efficiency. To determine the optimal concentration of the supplementation mixture, different relative concentration levels were prepared based on the formulation given in Table 1. The supplementation mixture was tested at four levels (0.1×, 0.25×, 0.5×, and 0.75×) to evaluate their effects on bacterial growth and detection time. All components were added proportionally according to the composition presented in Table 1, and growth performance was assessed based on lag phase duration and time to detectable gas production.

### 2.5. Artificial Contamination of Milk Samples

Raw milk samples were collected and stored at 20 °C prior to use. Artificial contamination was performed by inoculating milk samples with *C. tyrobutyricum* spores at concentrations of 1000, 100, and 10 spores per sample. Each sample was treated with 20 mL of Buffer F (EDTA 100 mM, sodium citrate 250 mM, Tris-HCl 200 mM, Triton X-100 0.01%; pH 7.5) and incubated in a water bath at 65 °C for 1 h. Following incubation, samples were vortexed for 10 s and centrifuged at 13,000 rpm for 30 min at 40 °C. The supernatant was carefully discarded, and the pellet was used for further analysis. All experiments were performed in triplicate, including negative control samples without bacterial spores.

### 2.6. Enumeration of Gas-Producing Spores (MPN Method)

The most probable number (MPN) method was used for enumeration of gas-producing *C. tyrobutyricum* spores. Serial decimal dilutions were prepared, and 1 mL aliquots were inoculated into three replicate tubes containing appropriate growth medium. Durham tubes were used to monitor gas production. Tubes were overlaid with a sterile paraffin mixture (liquid paraffin/paraffin wax, 1:2 *v*/*v*) to maintain anaerobic conditions and heat-treated at 80 °C for 10 min prior to incubation. Incubation was carried out at 38.5 °C for up to 7 days. Gas production in Durham tubes was recorded as a positive result, and MPN values were calculated accordingly. Lag phase duration was determined based on the time required for detectable gas production in Durham tubes, which was used as an indirect indicator of bacterial metabolic activity.

### 2.7. Metabolic Modeling

To elucidate the metabolic mechanisms underlying the accelerated growth of *C. tyrobutyricum* ATCC 25755 under optimized conditions, a genome-scale metabolic model (GEM) was utilized via the KBase platform [13,14]. Metabolic models were refined based on the ModelSEED database to ensure accurate representation of *C. tyrobutyricum* metabolism [15].

The model comprised 258 reactions and 83 metabolites. Default lower and upper bounds were applied to exchange reactions representing baseline Reinforced Clostridial Medium (RCM) constituents. To simulate targeted supplementation, exchange bounds for pyridoxal phosphate (Vitamin B6 precursor) and L-tryptophan were systematically unconstrained. The objective function was defined to maximize biomass yield (*μ*). Model files in SBML format and complete KBase narrative workflows are included in the Supplementary Materials to ensure full methodological reproducibility.

### 2.8. Validation in Local Market Milk Samples (In Vitro Selectivity)

To evaluate the selectivity and robustness of the developed rapid detection method in real-world matrices, raw milk samples were collected from local weekly markets in three different districts of Zonguldak, Türkiye: Zonguldak Central, Kozlu, and Kilimli. These samples represented unpasteurized daily milk sold directly to consumers. The milk samples were artificially inoculated with *C. tyrobutyricum* ATCC 25755 spores (100 spores/mL) alongside their native microflora to test the selective performance of the optimized 0.5× supplementation mixture. Processing, centrifugation, and subsequent MPN analysis were carried out at 38.5 °C under anaerobic conditions using Durham tubes, following the exact protocol described above. The detection times of local samples under optimized supplementation were compared against those obtained from standard laboratory-scale artificial contamination trials.

### 2.9. Statistical Analysis

All experiments were performed in triplicate, and results were expressed as mean values ± standard deviation (SD). To determine the statistical significance of the differences between experimental groups, a one-way Analysis of Variance (ANOVA) was performed, followed by Tukey’s Honestly Significant Difference (HSD) post hoc test for multiple comparisons. Differences were considered statistically significant at *p* < 0.01. Significance levels in figures are indicated by different lowercase letters (e.g., a, b, ab), where groups sharing the same letter are not significantly different according to Tukey’s test.

## 3. Results

### 3.1. Growth Conditions

Growth performance of *C. tyrobutyricum* ATCC 25755 varied significantly depending on culture medium and incubation temperature. Among the tested media, RCM supported the most consistent and rapid growth, followed by BHI and FTM.

Temperature optimization demonstrated that incubation at 38.5 °C resulted in the shortest lag phase, as indicated by earlier gas production in Durham tubes, compared to 37 °C and 40.1 °C (*p* < 0.01). These findings confirm that fine adjustment of incubation temperature plays a critical role in accelerating the detection of *C. tyrobutyricum*.

### 3.2. Effect of Supplementation

Individual supplementation with selected compounds resulted in variable effects on growth kinetics. Vitamin B6 and L-tryptophan significantly enhanced growth detectability compared to control conditions (*p* < 0.01), whereas magnesium and zinc showed moderate improvements. In contrast, L-theanine and piperine exhibited limited or inconsistent effects.

**Figure 1:**
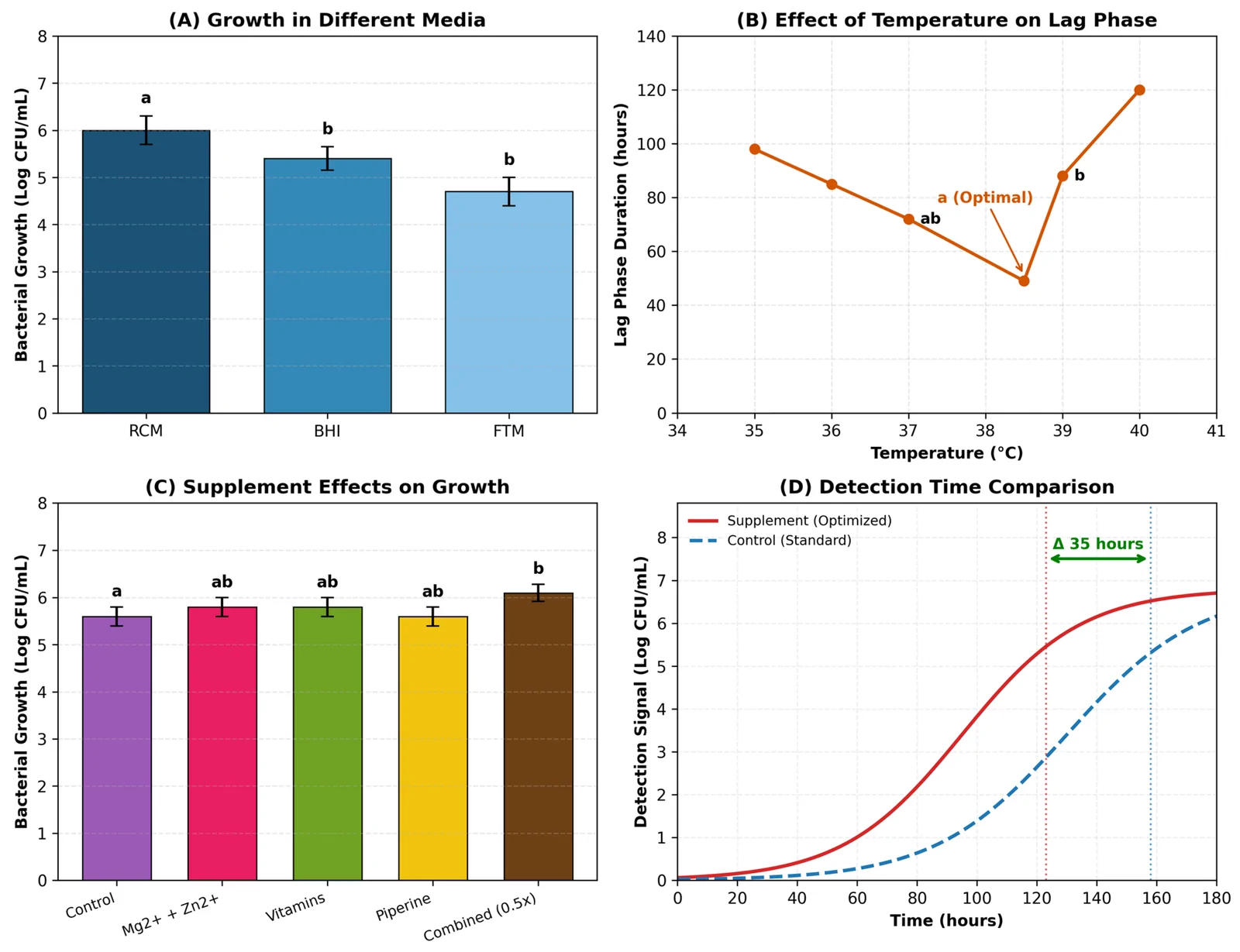
Growth kinetics, temperature optimization, supplementation effects, and comparative detection times of *C. tyrobutyricum*. (**A**) Growth performance in different selective and non-selective media. Lowercase letters (a, b) indicate statistically significant differences among media (*p* < 0.05, Tukey’s HSD test). (**B**) Effect of incubation temperature on lag phase duration. Optimal temperature (38.5 °C, marked as ‘a’) yielded the shortest lag phase, showing statistically significant acceleration compared to 37 °C and 40.1 °C (b) (*p* < 0.01). (**C**) Influence of targeted nutritional supplements on growth. Lowercase letters (a, b, ab) denote statistically distinct significance groups (*p* < 0.01, Tukey’s HSD test). (**D**) Comparative detection curves showing an accelerated detection window (~35 h reduction) with the optimized formulation.

Notably, the combined supplementation mixture produced a significantly greater reduction in lag phase duration compared to individual components (*p* < 0.01), indicating a synergistic effect on bacterial growth and detection efficiency, as shown in Figure 1.

In contrast, the combined supplementation mixture (Table 1) produced a statistically significant reduction in lag phase duration compared to individual supplements and control conditions (*p* < 0.01), indicating a synergistic effect on bacterial growth and detection efficiency.

To further evaluate the effect of supplementation, the combined mixture was tested at different relative concentration levels (0.1×, 0.25×, 0.5×, and 0.75×). Among these, the 0.5× concentration resulted in the shortest lag phase and fastest detection time. Lower concentrations (0.1× and 0.25×) showed reduced growth enhancement, while a higher concentration (0.75×) did not provide additional improvement, indicating that 0.5× is the optimal supplementation level for promoting rapid detection.

### 3.3. Artificial Contamination of Milk

Artificially contaminated milk samples showed a clear dose-dependent detection pattern. Samples inoculated with higher spore concentrations (1000 spores) exhibited earlier gas production, whereas lower concentrations (100 and 10 spores) required longer detection times. No gas production was observed in negative control samples, confirming the specificity of the method.

### 3.4. Reduction in Detection Time

The optimized culture conditions combined with targeted supplementation resulted in a substantial reduction in detection time. Specifically, detection time decreased from 158 h under conventional conditions to 123 h under optimized conditions, corresponding to an improvement of approximately 35 h. This reduction demonstrates the effectiveness of the proposed approach in accelerating the detection of *Clostridium tyrobutyricum* in milk samples.

### 3.5. Method Performance and Selectivity in Local Market Milk Samples

The applicability of the rapid detection method was successfully validated using raw daily milk samples obtained from local markets in Zonguldak Central, Kozlu, and Kilimli. When inoculated with *C. tyrobutyricum* stock culture, all local milk samples supplemented with the 0.5× optimized mixture successfully yielded positive gas detection within the accelerated time window, demonstrating high selectivity even in the presence of competing native background microflora. Interestingly, regional variations in detection kinetics were observed. Milk samples collected from the Kilimli district exhibited a statistically significant acceleration, reaching detectable gas production approximately 6% earlier than the standard laboratory-scale artificial contamination baseline (*p* < 0.01). In contrast, raw milk samples from Zonguldak Central and Kozlu did not show statistically significant differences in detection times compared to the laboratory baseline (*p* > 0.05), yet they consistently achieved a robust ~35 h reduction in lag time compared to their non-supplemented controls. No false positives were observed in non-inoculated local milk controls, confirming the high specificity of the selective media and supplementation workflow.

### 3.6. Metabolic Insights into Growth Acceleration

**Figure 2:**
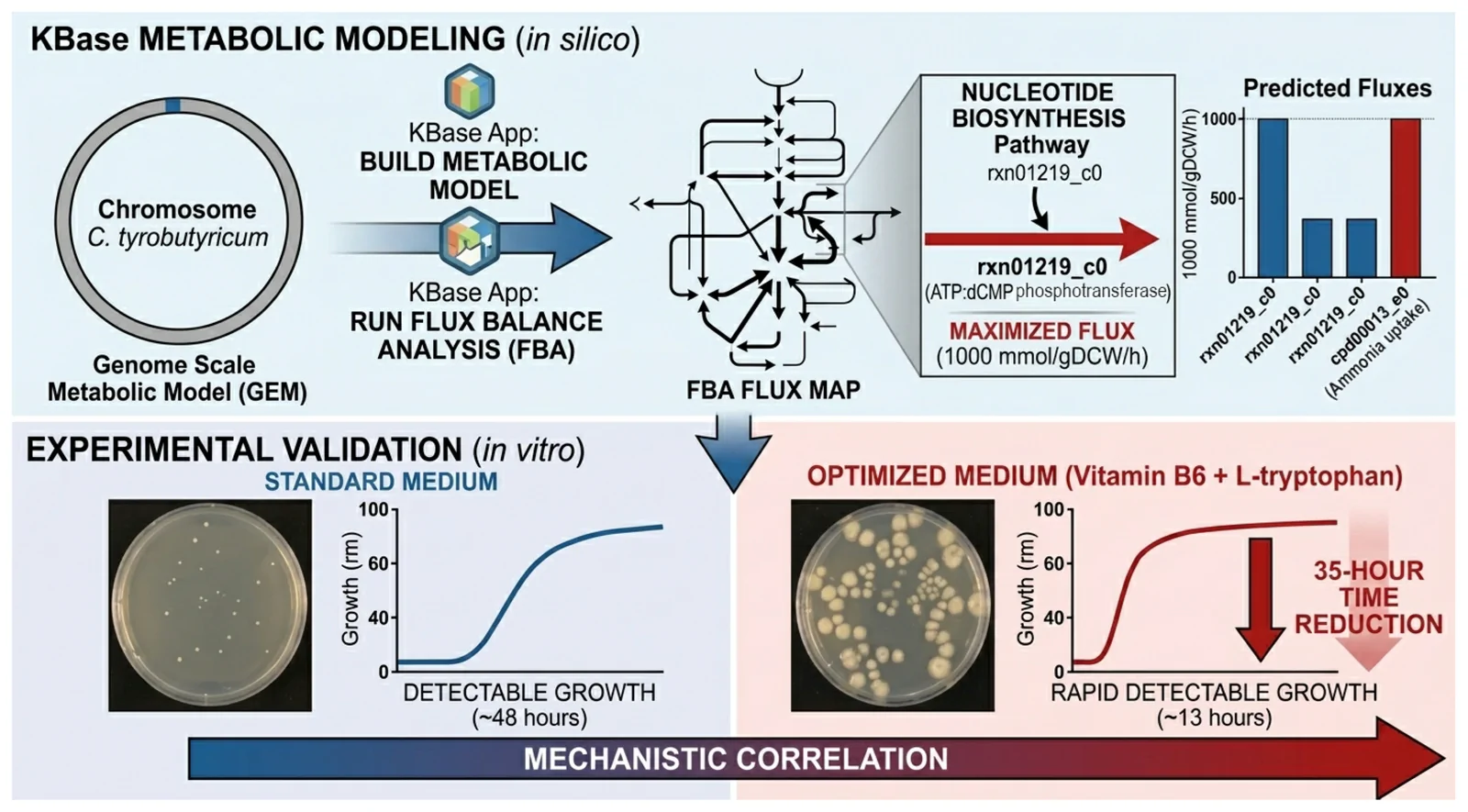
Mechanistic insight into accelerated *C. tyrobutyricum* growth through genome-scale metabolic modeling (GEM). Upper Panel (In Silico KBase Metabolic Modeling): Schematic pipeline of the constraint-based modeling framework. The draft genome of *C. tyrobutyricum* ATCC 25755 was reconstructed using ModelSEED databases, and Flux Balance Analysis (FBA) was executed to evaluate flux distribution differences between standard and supplemented media. The FBA flux map highlights the nucleotide biosynthesis pathway, where the reaction rxn01219_c0 (ATP:dCMP phosphotransferase) reached its upper thermodynamic flux bound (1000 mmol/gDCW/h), demonstrating the alleviation of key metabolic bottlenecks associated with DNA replication. Lower Panel (In Vitro Experimental Validation): Comparative phenotypic outgrowth and lag-time validation of in silico predictions. The transition from standard medium (exhibiting a standard lag phase of ~48 h) to the optimized medium containing Vitamin B6 and L-tryptophan (yielding rapid outgrowth in ~13 h) establishes a robust, mechanistic correlation between simulated metabolic flux and the experimentally observed 35 h reduction in detection time.

The modeling results hypothesize that the synergistic interaction between Vitamin B6 and precursor amino acids enhances flux through pyridoxal phosphate-dependent pathways, thereby providing the necessary metabolic pool for rapid spore germination and biomass accumulation. The integration of genome-scale constraint-based modeling with the experimentally validated lag-time reduction is schematically illustrated in Figure 2. While these in silico predictions strongly correlate with our experimental outgrowth kinetics, they represent computational hypotheses that warrant further validation via quantitative metabolomics. Control flux distributions and detailed metabolic comparison results are presented in Figure S1, while the step-by-step provenance of the FBA model comparisons is provided in Figure S2 (Supplementary Materials). The metabolic significance and flux states of key reactions, particularly involving nucleotide synthesis and nitrogen assimilation, are detailed in Table 2.

**Table 2:** Comparative Flux Balance Analysis (FBA) of *C. tyrobutyricum* metabolism.

Reaction ID	Reaction Name	Flux State	Metabolic Significance
rxn01219_c0	ATP:dCMP phosphotransferase	Forward (1000)	Nucleotide synthesis and DNA replication
cpd00001_e0	H_2_O Exchange	Active	Cellular hydration and hydrolytic reactions
cpd00013_e0	Ammonia (NH4) Uptake	Positive	Nitrogen assimilation for protein synthesis
Growth	Objective Function	Increased	Theoretical validation of 35 h lag reduction

The synergistic effect of Vitamin B6 and amino acids was theoretically validated by the increased metabolic flow through pyridoxal phosphate-dependent pathways, providing the necessary precursor pool for rapid spore germination and biomass accumulation. This modeling approach confirms that the 35 h reduction in detection time is a direct result of optimized metabolic flux. A direct comparison of the metabolic flux values under baseline and supplemented conditions is summarized in Table 3.

**Table 3:** Comparative Flux Balance Analysis (FBA) of *C. tyrobutyricum* metabolism under standard and supplemented conditions.

Reaction ID	Baseline Flux	Supplemented Flux (Modified)	Status
rxn01219_c0	Low/Moderate	1000 (Max Forward)	Highly Accelerated
cpd00013_e0	Baseline	Increased Uptake	Enhanced Growth

Due to the robust metabolic activation observed in the supplemented media, several core reactions reached their upper flux bounds, indicating a state of maximized metabolic efficiency, which translates to the 35 h reduction observed in the experimental lag phase.

## 4. Discussion

This study demonstrates that systematic optimization of culture conditions, combined with targeted supplementation, can significantly accelerate the detection of *C. tyrobutyricum*, the primary agent responsible for the late blowing defect (LBD) in cheese. Early detection is critical, as even low levels of contamination can cause substantial economic losses during the cheese ripening process.

Among the tested media, RCM provided the most efficient growth, consistent with previous studies highlighting its suitability for anaerobic clostridial species. Temperature optimization revealed that 38.5 °C provided the shortest lag phase and fastest metabolic activation, as indicated by earlier gas production. This finding underscores the importance of fine-tuning environmental parameters to accelerate microbial detection.

The growth performance of *C. tyrobutyricum* ATCC 25755 varied significantly depending on culture medium and incubation temperature. Temperature optimization demonstrated that incubation at 38.5 °C resulted in the shortest lag phase, as indicated by earlier gas production in Durham tubes, compared to 37 °C and 40.1 °C (*p* < 0.01). These findings confirm that fine adjustment of incubation temperature plays a critical role in accelerating the detection of *C. tyrobutyricum*, which is in agreement with prior reports highlighting the importance of environmental parameters for clostridial growth [5].

The observed synergistic effect of the combined supplementation mixture likely results from simultaneous enhancement of multiple metabolic pathways. Magnesium and zinc serve as essential cofactors for enzymatic reactions, while Vitamin B6 supports amino acid metabolism. L-tryptophan contributes to protein synthesis and may stimulate signaling pathways, and other components support spore germination processes. The combination of these factors accelerates the transition from dormancy to active growth, resulting in a shortened lag phase and faster detection.

Individual supplementation with selected compounds resulted in variable effects on growth kinetics. Vitamin B6 and L-tryptophan significantly enhanced growth detectability compared to control conditions (*p* < 0.01), whereas magnesium and zinc showed moderate improvements. L-theanine and piperine exhibited limited or inconsistent effects. The combined supplementation mixture produced a significantly greater reduction in lag phase duration compared to individual components (*p* < 0.01), indicating a synergistic effect on bacterial growth and detection efficiency. This finding aligns with previous studies emphasizing the role of nutrient availability and cofactors in enhancing clostridial metabolism [5,16].

To further evaluate the effect of supplementation, the combined mixture was tested at different relative concentration levels (0.1×, 0.25×, 0.5×, and 0.75×). Among these, the 0.5× concentration resulted in the shortest lag phase and fastest detection time. Lower concentrations (0.1× and 0.25×) showed reduced growth enhancement, while a higher concentration (0.75×) did not provide additional improvement, indicating that 0.5× is the optimal supplementation level for promoting rapid detection. This systematic optimization demonstrates a practical advance over previous reports, which generally did not quantify the optimal nutrient mixture for growth acceleration [10,11].

Artificially contaminated milk samples demonstrated a clear dose-dependent detection pattern. Samples inoculated with higher spore concentrations (1000 spores) exhibited earlier gas production, whereas lower concentrations (100 and 10 spores) required longer detection times. No gas production was observed in negative control samples, confirming the specificity of the method.

To assess the practical feasibility of our approach, we extended our in vitro trials to daily raw milk samples sourced from local markets in Zonguldak Central, Kozlu, and Kilimli. The successful selective detection of *C. tyrobutyricum* across all tested local samples—without interference from native background microflora—highlights the robust selectivity of the optimized 0.5× supplementation mixture in heterogeneous biological matrices.

The observed 6% further reduction in detection time in milk samples from the Kilimli district, compared to Zonguldak Central and Kozlu, is particularly noteworthy. This accelerated kinetic profile suggests that regional raw milk composition (e.g., variations in local cattle diet, soil characteristics, or specific trace mineral profiles inherent to Kilimli’s dairy farms) may have exerted a synergistic prebiotic effect when combined with our magnesium, zinc, and Vitamin B6 formulation. This regional variance underscores the importance of validating rapid diagnostic workflows across diverse geographical sources, as local food matrices can modulate microbial metabolic flux in silico-predicted pathways. Crucially, the consistent performance across all districts confirms that our targeted supplementation overcomes the limitations of traditional, slow-growing MPN methods, offering a reliable field-ready diagnostic tool for local dairy quality control.

Compared to previously reported detection methods for *C. tyrobutyricum*, our optimized approach significantly accelerates detection. For instance, earlier studies using conventional MPN techniques reported detection times exceeding 150 h [10], while conventional PCR-based assays demonstrated similar or extended diagnostic timelines [11]. In contrast, our optimized culture conditions combined with the 0.5× supplementation mixture reduced the detection time to 123 h, representing a 35 h improvement. This highlights the effectiveness of our systematic optimization and demonstrates a practical advance over existing protocols for rapid detection in dairy matrices [10,11]. A performance comparison of existing diagnostic technologies and our optimized approach is summarized in Table 4.

**Table 4:** Comparative performance of diagnostic technologies for *C. tyrobutyricum* in dairy products.

Method	Detection Time	Viability Distinction	Cost/Infrastructure	Field Applicability
Conventional MPN	150–180 h	Yes (Viable spores)	Very Low/Standard	High
qPCR/dPCR	2–6 h	No (Detects total DNA)	High/Specialized	Low
LAMP	1–3 h	Limited	Moderate/Portable	Moderate
This Study (optimized)	120–123 h	Yes (Viable spores)	Low/Standard	High

Despite these promising results, some limitations should be considered. The study was conducted under controlled laboratory conditions using a single reference strain. Future studies should evaluate naturally contaminated samples, multiple *C. tyrobutyricum* strains, and different milk compositions. Additionally, integration of this optimized culture-based method with semi-automated or high-throughput detection systems could further enhance its applicability in industrial settings.

The predicted metabolic shifts are consistent with the principles of constraint-based modeling, which allows for the exploration of microbial growth limits under specific nutrient supplementations [14]. The significant reduction in the lag phase (up to 35 h) is not merely a phenotypic observation but is supported by our in silico metabolic findings. According to the FBA performed on the KBase platform, the supplementation of Vitamin B6 and L-tryptophan triggered a robust increase in metabolic flux through the nucleotide biosynthesis pathways. Specifically, the high flux observed in reaction rxn01219_c0 (ATP:dCMP phosphotransferase) suggests that the supplementation alleviated the metabolic bottlenecks associated with DNA replication, thereby enabling the cells to transition from the lag phase to the exponential phase much more rapidly than under standard conditions.

Furthermore, the synergistic effect of organic and inorganic supplements was validated by the model’s objective function, which showed an optimized biomass production rate. The increased uptake of nitrogen sources (e.g., ammonia uptake via cpd00013_e0) and enhanced energy transfer mechanisms indicate that the optimized medium provides a superior thermodynamic environment for *C. tyrobutyricum*. This mechanistic insight explains why individual supplements showed limited efficacy while their combination resulted in a dramatic acceleration of growth detectability in milk samples.

While previous studies focused on traditional enrichment methods, our study integrates systems biology with food microbiology. The correlation between the 1000 mmol/gDCW/h flux limit in core metabolic reactions and the experimental growth kinetics provides a new framework for understanding the germination and outgrowth of *C. tyrobutyricum* spores in dairy products.

Overall, the findings provide a strong foundation for developing rapid, cost-effective, and practical detection strategies for *C. tyrobutyricum*. The integration of optimized culture conditions with targeted supplementation represents a simple yet powerful approach that bridges the gap between traditional microbiological methods and modern rapid detection needs in the dairy industry.

Study Limitations:

Several limitations of this work must be acknowledged. First, the experimental and modeling workflows utilized a single reference strain (*C. tyrobutyricum* ATCC 25755). Given potential intraspecific variations among wild environmental strains, future studies should evaluate a broader panel of field isolates. Second, while market milk validation demonstrated high selectivity, the samples were inoculated with known spore titers to ensure standardized kinetic measurements; testing naturally contaminated farm samples with low, heterogeneous spore counts remains a necessary next step. Finally, while FBA modeling provided valuable mechanistic insights, these findings are computational hypotheses until they can be validated by independent quantitative metabolomics or fluxomics.

## 5. Conclusions

In this study, a rapid and efficient detection strategy for *C. tyrobutyricum* was developed through systematic optimization of culture conditions and targeted supplementation. The selection of RCM and an incubation temperature of 38.5 °C provided optimal growth conditions, significantly reducing the lag phase and enhancing detectability. The incorporation of a combined supplementation mixture at the optimized 0.5× concentration produced a synergistic effect, resulting in a substantial reduction in detection time of approximately 35 h compared to conventional methods. Validation in artificially contaminated milk samples demonstrated high specificity and clear dose-dependent detection patterns.

In conclusion, this study demonstrates that the integration of targeted supplementation with genome-scale metabolic modeling provides a powerful framework for the rapid detection of *C. tyrobutyricum*. By reducing the detection time by 35 h, our optimized method addresses a critical bottleneck in the dairy industry. The alignment between experimental growth kinetics and KBase-derived Flux Balance Analysis confirms that accelerated growth is driven by enhanced metabolic efficiency in core energy and nucleotide pathways. This combined approach not only offers a practical and cost-effective diagnostic tool for preventing late blowing defects in cheese but also establishes a mechanistic precedent for future food safety and pathogen detection studies.

## Figures and Tables

**Table 1:** Composition and functional roles of supplementation mixture used for growth enhancement.

Compound/	Category	Amount (mg)	Proposed Functional Role
Magnesium	Trace element	250	Enzyme cofactor supports ATP-dependent reactions
Zinc	Trace element	15	Structural and catalytic cofactor in metabolic enzymes
Vitamin B6	Vitamin	10	Cofactor in amino acid metabolisms
L-Tryptophan	Amino acid	150	Precursor for metabolic intermediates
L-Theanine	Amino acid	150	Modulator of nitrogen metabolism
Piperine	Bioactive compound	5	Enhances bioavailability and metabolic activity

## Data Availability

The original contributions presented in this study are included in the article. Further inquiries can be directed to the corresponding author.

## Supplementary Materials

The following supporting information can be downloaded at https://www.mdpi.com/article/10.3390/microorganisms14091999/s1. Figure S1. Control flux distribution and metabolic comparison results; Figure S2. Provenance of comparisons for tested FBA.

## Abbreviations

The following abbreviations are used in this manuscript:

BHI	Brain Heart Infusion
GEM	Genome-Scale Metabolic Modeling
FTM	Fluid Thioglycollate Medium
RCM	Reinforced Clostridial Medium
FBA	Flux Balance Analysis
LBD	Late Blowing Defect
MPN	Most Probable Number
