# Supplementary material for: Integrating Metabolic Modeling and Targeted Supplementation for the Rapid Detection of Clostridium tyrobutyricum in Dairy Products
Source: Microorganisms. 2026 Sep 9;14(9):1999. doi: 10.3390/microorganisms14091999 (PMC13609277; doi:10.3390/microorganisms14091999)
Supplement: Supplementary file 1 [file microorganisms-14-01999-s001.zip › microorganisms-4476500-supplementary.pdf]

Integrating Metabolic Modeling and Targeted Supplementation for the Rapid Detection of *Clostridium tyrobutyricum* in Dairy Products

Idris Arslan*

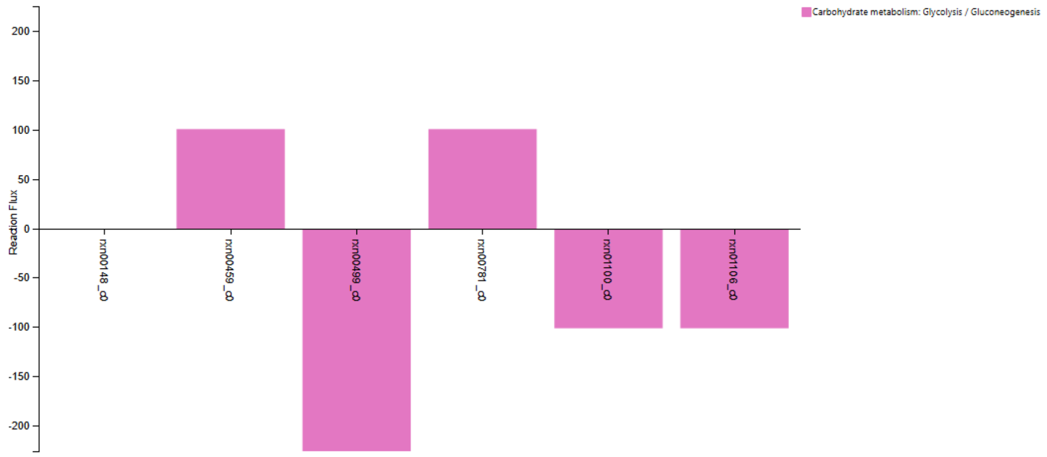

Figure S1. Control flux distribution and metabolic comparison results

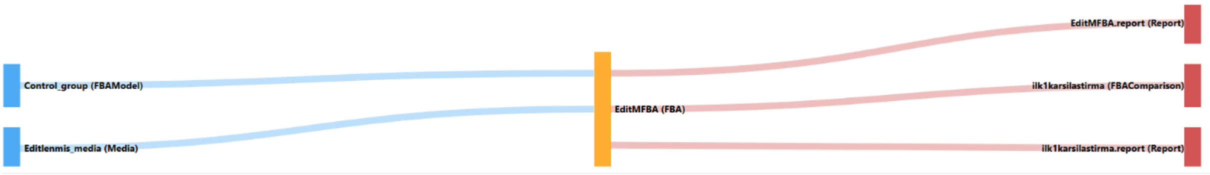

Figure S2. Provenance of comparision of FBA tested
